# Progress in Neurology

**Published:** 1903-05-30

**Authors:** 


### Progress in Neurology.

(*Continued from page* 136.)

**Epilepsy.**—In an article on the problem of epilepsy Clark and Prout say that the excitant is probably a toxic or autotoxic poison, for the following reasons: The lesion is a general one involving the entire cortex, but most extensively the cells of the second or so-called pyramidal layer which present a relatively larger surface exposure to the circulating poison in proportion to their volume than the large pyramids. There is a marked neuroglial proliferation. And lastly the changes in the cell are quite analogous with those definitely known to be caused by toxic agents such as furfurol, alcohol, and the tetanus bacillus and its toxins. Microscopical examination of the brain from numerous cases shows that the same lesions are uniformly present, differing only in degree, being most marked in patients who had died in status epilepticus. The nucleus of the cell was swollen, with destruction of the nuclear membrane and intranuclear network, and the nucleolus was quite loose. The primary lesion is thus a cell destruction. The secondary one is a sclerosis more or less diffuse of various cortical areas. It is to be remarked that quite similar changes are found in myoclonus, general paresis and chorea; the lesion is not confined to epilepsy. In opening a discussion on the treatment of epilepsy, Russell[5] said that since we are still in ignorance of the essential cause of epilepsy, in spite of all the researches which have been made, no really rational treatment had been introduced. Of the theories advanced to explain its occurrence, that which supposes it to be due to an autointoxication had received most acceptance, though it could not be said to be proved. The bromides are still the most potent agents in treatment. There is no special advantage in giving the mixed potassium, sodium, and ammonium bromides, or in the use of strontium bromide. Bromide of camphor gives excellent results. Whichever salt is given should be administered an hour before bedtime in nocturnal epilepsy, and two hours before any diurnal attack is due. Borax, belladonna, digitalis, and arsenic are often added with advantage. Flechsig's opium method has little to recommend it, though a subcutaneous injection of morphia is one of the surest methods of arresting the status epilepticus. It is better, however, to rely on the subcutaneous use of hyoscine in this condition. All stimulants, including tea and coffee, should be forbidden, and only a small amount of meat should be allowed. The most important modification introduced of recent years in the dietetic treatment of epilepsy is the exclusion of ordinary salt from the food; if this be done, much smaller doses of bromide are effectual. Horsley suggested that epilepsy should be classified into idiopathic (which is localised or generalised), Jacksonian (which is traumatic, congenital, or neoplastic), reflex, and hystero-epilepsy. In generalised idiopathic and hystero-epilepsy operative measures are useless; they are most likely to cure localised Jacksonian cases, whether due to trauma or neoplasm.

**Myoclonus Epilepsy.**—Pierce Clark and Prout[6] have added three cases to the scanty records of this rare condition and given a summary of the existing state of our knowledge. The predisposing causes are family degeneracy, marked by alcoholism, tuberculosis, epilepsy, insanity, and chorea. In the development of the disease epilepsy appears first in one-half of the cases, myoclonus first in a third, whilst in the remainder the two diseases have a simultaneous onset. As a rule the epilepsy continues throughout life; the seizures are not preceded by a sensory aura, but premonitory signs of increased myoclonic contractions are in evidence in the great majority. Periods of long remission from the epileptic seizures may occur under treatment, and no myoclonus-epileptic is reported as dying of the status epilepticus. The myoclonus contractions—the great characteristic of which is that there is no loss of consciousness—are usually lightning-like or may have a fibrillary character involving parts of certain muscles only. The clonic contractions of myoclonus may end imperceptibly in the tonic stage of the epileptic paroxysm. The myoclonus contractions are often strong and affect large masses of muscles, and as a consequence locomotive effect is common. It is comparatively rare for myoclonus-epileptics to pass days entirely free from myoclonus, and as the malady develops the myoclonus becomes more or less persistent during the waking period and may thus cause death. Under bromide treatment, however, not merely the epilepsy but the myoclonus also may undergo entire remission for long periods. The myoclonus is absent during sleep, and in the early stages patients are able to check the myoclonic contractions by an effort of the will. The mental state of myoclonus-epileptics varies from slight mental enfeeblement to a more or less complete imbecility. The prognosis of the affection as to recovery is always poor, and treatment can be only palliative. Bromides stand in the first place. Microscopical examination of the cerebral cortex of these cases shows the lesion of epilepsy in the second layer cells —*i.e.* destruction of the intranuclear network and its replacement by a granular substance—plus a maximum intensity of the same form of cell-death in the large pyramidal cells of the third layer. Presumably the latter changes underlie the myoclonic spasm. The lesion of myoclonus-epilepsy thus appears to be in the cerebral cortex involving the nucleus and the intranuclear network of cells of both sensory and motor types. Its pathogenesis is probably brought about by a faulty chemotaxis of these cells because of an inherent organic anomaly. Whilst each condition—epilepsy and myoclonus—maintains its separate morbid entity, the two are closely allied and often found associated clinically and pathologically.

**Aphasia of Uræmic Origin.**—Riesman[7] has reported two cases of aphasia of uræmic origin. In the first, the patient, a man aged 42 years, suffered from attacks of transient right-sided hemiplegia and aphasia, with no loss of consciousness or anæsthesia. He could not speak during the attacks, but could understand what was said and could recognise his surroundings. His arteries were thickened, and there were both polyuria and albuminuria. He finally died in coma. In the second case, a woman aged 46 years, the transient attacks of aphasia and agraphia were not accompanied by any paralysis. The duration of such attacks of aphasia from uræmia, in these and other patients, has varied from a fleeting attack of five minutes' duration to one lasting a week, but the average period was from 24 to 48 hours. In one-half of the cases there was an accompanying motor paralysis. The aphasia was of the motor variety, whilst word-blindness or word-deafness was quite rare. In several cases venesection was performed, and excellent results followed. In cases which ended fatally there was no gross lesion or destruction of brain tissue to account for the aphasia or hemiplegia, the only lesions present being cerebral congestion and œdema.

[4] Med. Rec., Feb. 14, 1903. [5] Brit. Med. Jour., Feb. 14, 1903 [6] Amer. Jour. Insanity, Oct. 1902. [7] Jour. of Amer. Med. Assoc., Oct. 11, 1902.

*(To be continued.)*

